# Retraction Note: Alpha-synuclein overexpression in the olfactory bulb initiates prodromal symptoms and pathology of Parkinson’s disease

**DOI:** 10.1186/s40035-022-00310-4

**Published:** 2022-06-29

**Authors:** Haichen Niu, Lingyu Shen, Tongzhou Li, Chao Ren, Sheng Ding, Lei Wang, Zhonghai Zhang, Xiaoyu Liu, Qiang Zhang, Deqin Geng, Xiujuan Wu, Haiying Li

**Affiliations:** 1 Department of Genetics, Xuzhou Medical University, Xuzhou, 221004 China; 2 Department of Epidemiology and Health Statistics, Xuzhou Medical University, Xuzhou, 221004 China; 3 Department of Neurology, Affiliated Yantai Yuhuangding Hospital of Qingdao University, Yantai, 264000 China; 4 College of Medicine, Institute for Behavioral Medicine Research, The Ohio State University, Columbus, OH USA; 5 Department of Neurology, Affiliated Hospital of Xuzhou Medical University, Xuzhou, 221004 China; 6 Department of Pathology, Xuzhou Medical University, Xuzhou, 221004 China

## Retraction Note: Translational Neurodegeneration (2018) 7:25 10.1186/s40035-018-0128-6

The Editor-in-Chief has retracted this article. After publication, concerns were raised regarding image overlap across various panels in Fig. 8. The authors have stated that incorrect images were used, but have been unable to provide the full raw data. Additionally, the authors could not justify the anesthetic protocol used for rat stereotaxic surgery. The Editor-in-Chief therefore no longer has confidence in the presented data and ethics of this article.

Author Haichen Niu has stated on behalf of all co-authors that they agree to this retraction.

